# Development of a justification process for selecting alternative risk reduction measures

**DOI:** 10.3389/fpubh.2025.1600801

**Published:** 2025-06-25

**Authors:** Pavlo Saik, Vitalii Tsopa, Olena Yavorska, Serhii Cheberiachko, Mariia Brezitska, Andrii Yavorskyi, Vasyl Lozynskyi

**Affiliations:** ^1^Belt and Road Initiative Center for Chinese-European Studies (BRICCES), Guangdong University of Petrochemical Technology, Maoming, China; ^2^MIM-Kyiv, Kyiv, Ukraine; ^3^Dnipro University of Technology, Dnipro, Ukraine

**Keywords:** professional disease, risk, pneumoconiosis, safety, occupational risks

## Abstract

**Introduction:**

The aim of this study is to develop a process for determining a set of alternative preventive measures to reduce risk levels, using the example of reducing the incidence of occupational pneumoconiosis in miners under conditions of financial cost minimization.

**Methods:**

This study was conducted using a system analysis to identify priority directions for risk reduction under limited financial resources based on the relationship between the measures and their risk reduction efficiency. A methodology was developed to justify the selection of risk reduction measures from a set of alternatives based on the calculation of an efficiency factor grounded in financial losses. An eleven-step risk management process was designed to determine alternative preventive measures, characterized by feedback loops that enable the selection of optimal risk reduction strategies.

**Results:**

This study presents algorithms for solving three types of decision-making problems regarding the selection of combinations of preventive measures from a defined set of alternatives. These algorithms consider the priority of the measures and allow for a comparison between the effectiveness of several measures and a single measure that can also reduce the risk to an acceptable level.

**Discussion:**

An example of calculating the efficiency of preventive measures to reduce the risk of pneumoconiosis among miners is also provided. A key feature of this study is the improvement in the risk management process by integrating the efficiency factor of risk reduction under financial constraints.

## 1 Introduction

The processes of risk management at a production site are the basis for decision-making in various production spheres (1). This helps ensure the functional stability of any organization under changing conditions (2). Most often, the Plan-Do-Check-Act (PDCA) approach is used for risk management (3). It includes the following: a planning process that identifies possible hazards, threats, or inconsistencies with the assessment of consequences when hazardous events occur; an action when preventive and protective measures (henceforth, measures) are substantiated based on the risk level assessment; verification, i.e., substantiation of the number of measures to reduce risks due to a specified hazard, based on, for instance, financial costs; and implementation, i.e., introduction of the selected measures into the production process. The PDCA approach ensures the implementation of planning, resource management, product implementation and measurement, and continuous improvement (4, 5). To support the unified global implementation of risk management, the ISO 31000 standard was developed. It is a simple way to apply risk-oriented thinking while solving problems to improve PDCA implementation in all types of organizations (6).

This represents a simple way to apply risk-oriented thinking to improve the implementation of the PDCA cycle across all types of organizations (6). Its application enables structuring approaches to determine the residual risk, which are often established based on the “as low as reasonably practicable” (ALARP) principle (7). This gives rise to the task of evaluating the feasibility (efficiency) of applying all possible and available alternatives to control and reduce risks (8). The efficiency of risk control alternatives is typically assessed by a group of experts based on their impact on financial costs, reputational losses, implementation time, and achievement of strategic goals (9, 10). This process is often conducted using questionnaires (10), surveys (11), and various models to evaluate the effectiveness of preventive risk reduction measures (12, 13). For instance, the Best-Worst Method (BWM) ranks alternatives based on their importance or estimated costs (14). The Analytic Hierarchy Process (AHP), which requires *n* (*n* − 1)/2 pairwise comparisons of defined alternatives, is also widely used along with the Stepwise Weight Assessment Ratio Analysis (SWARA) method (15). Therefore, it is essential to validate the proposed measures and adjust them based on their identified shortcomings (16–18).

Simultaneously, the considered methods do not allow the selection of a specific combination of alternatives for risk reduction. In some cases, a combination of several preventive measures may be more effective than a single risk control measure. This creates the relevant task of developing a process to justify the feasibility of selecting multiple alternative measures to reduce risks to an acceptable level.

Over the last decade, several methods have been developed and improved to support risk management processes that establish cause-and-effect relationships among hazards, hazardous events, and the consequences of hazardous events, making it possible to assess the risk levels of industrial activities (4). FMECA (19) and HAZOP are the most extensive and are described in detail by standards (9, 20). Induction and deduction methods or their combinations are implemented in the ETA and FTA approaches (21) as well as FAHP and FTOPSIS (22) are widely used to identify cause-and-effect problems. However, there is little scientific work to substantiate the efficiency of applying best practices for risk reduction (23). Because the field of risk management is currently actively developing, the volume of information requiring appropriate analysis for its use in a particular field is growing significantly, particularly for decision-making regarding the introduction of means to protect workers from various hazards (24). Some studies describe the entire risk management process; however, they are specific to certain industries, limiting their use in other branches. For example, Yazo-Cabuya et al. (25) described a comprehensive list of hazards associated with construction, and Zacchei and Molina (26) elaborated on a powerful classification of hazards and hazardous factors by type. Namian et al. (27) defined the best methods of managing hazards in the field of transportation. The effectiveness of hazard control tools in terms of financial costs is proposed to be evaluated by the amount of the organization’s stability in the case of hazardous event occurrence (28), the reliability check of the technological process (29), or through the fulfillment of all the requirements stated in the internal documents of the enterprise in the field of occupational safety (23). Simultaneously, each organization needs to develop appropriate strategies that will contribute to an increase in security levels (30). However, all the above-mentioned approaches require appropriate substantiation of financial costs to implement the best practices in comparison with the costs of liquidation of consequences and providing help to the injured (31). This is explained by the fact that measuring and determining the risk reduction efficiency by appropriate measures is inherently a rather complex process requiring a certain study of legislative requirements (32), as well as analysis of a significant range of health effects (27). In addition, it is important to ensure constant monitoring of problematic indicators because of the distant consequences of their effects (31).

A review of the scientific literature shows that existing approaches to the justification of preventive measures are philosophical in nature, meaning that safety is considered the highest priority. Therefore, the cost of safety measures is deemed irrelevant compared to the value of human health and life, or based on the ALARP principle, which defines a risk standard whereby the risk level must be reduced as reasonably practicable within technically and economically feasible limits (29, 33).

To select alternative measures for risk reduction, it is often recommended to apply the cost–benefit principle. In this case, each preventive measure is analyzed using indices such as cost-effectiveness analysis (CEA) or the gross cost to avert fatality (GCAF) (31). This highlights the need to develop simple methods to substantiate the selection of a set of alternative preventive or protective measures that reduce risk to an acceptable level while minimizing financial costs and evaluating their effectiveness.

The aim of this study is to develop a process for determining a set of alternative preventive measures to reduce risk levels, using the case of reducing the incidence of occupational pneumoconiosis in miners under financial constraints.

To achieve this goal, the following tasks must be completed:

- Develop a risk management process that prioritizes the maximum efficiency of risk reduction measures.
- Solve the problem of determining alternative preventive measures and selecting appropriate measures to reduce the risks to an acceptable level, based on the condition of the efficiency of risk reduction to an acceptable level, and minimizing financial costs.

Provide an example of selecting measures of risk reduction to an acceptable level from a set of alternatives (provided that financial costs are minimized) in a coal mine to reduce pneumoconiosis cases among miners.

## 2 Materials and methods

**Figure 1:**
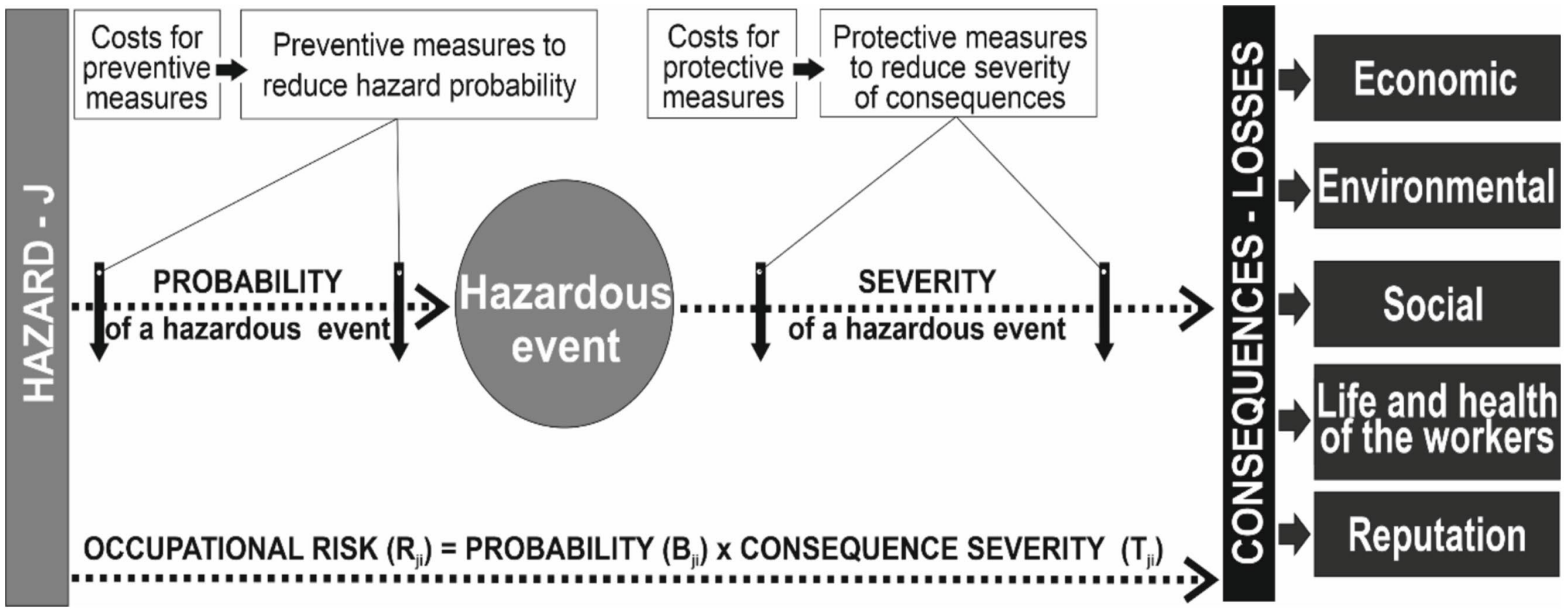
Risk management model taking into account measure costs.

This study was conducted using a systematic analysis to determine the priority directions for risk reduction in terms of financial resource limitations based on the relationship between the costs of implementing protective/preventive measures and their risk reduction efficiency (33). The basis of system analysis is a risk management model that reflects changes in the cause-and-effect relationship between a hazard and hazardous event, as well as the consequences of its occurrence. As a result, risk assessment makes it possible to determine and analyze the financial costs of measures to reduce risks to an acceptable level (Figure 1).

The risk management model also makes it possible to identify and analyze the number of errors, defects, or failures that may endanger productivity, reliability, safety (economic, environmental, and manufacturing), and occupational safety.

To solve the first problem regarding the development of a risk management process, taking into account the priority of the maximum effectiveness of risk reduction owing to risk reduction measures, based on the above model (Figure 1), 11 steps were proposed. Their consistent implementation will allow selecting those measures from the set of alternatives that will ensure risk reduction, provided they are effective.

In the first step of risk management, hazards, hazardous factors, and adverse consequences are identified (Step 1). This enables establishing cause-and-effect relationships between various components of the risk management model: “Hazard → Hazardous Event → Consequences.”

Subsequently, a risk analysis and assessment are conducted, where the risk level is defined as the product of the probability of a hazardous event and the severity of its consequences (Step 2). For this purpose, various Risk Assessment Matrices (RAMs) can be used. A RAM typically represents a two-dimensional grid: one axis contains categories of consequence severity, whereas the other axis contains categories of event probability (31, 34). Studies have shown that the most commonly used version is the 5 × 5 matrix (35).

However, such a matrix has several limitations, the most relevant of which is the difficulty in determining the exact risk level in the boundary zones (9). Therefore, it is more appropriate to apply more advanced matrices in which the risk level ranges are clearly defined. Based on this assumption, it is proposed for the purposes of the presented examples to consider the probability of a hazardous event as ranging from 0 to 1 and the severity of consequences as ranging from 0 to 100 points.

It is important to emphasize that this study focused on selecting alternative preventive and protective measures rather than substantiating the most accurate risk assessment method. Thus, using the widely accepted risk evaluation model (*R*) as the product of the probability of a hazardous event (*P*) and the severity of its consequences (*S*), i.e., *R* = *P* × *S*, aims to demonstrate the logic of the proposed process to substantiate the selection of alternatives.

It should also be noted that there is a clear need for simple and practical approaches to risk assessment that can be understood and applied directly to enterprises, even by personnel without deep knowledge of complex mathematical techniques.

In the third step, a set of possible alternative preventive and protective measures is determined to reduce the risk level, which is considered appropriate for the analyzed production process. This step takes into account the systemic interrelation that establishes the cause-and-effect chain between the components of the system: “Hazard → Hazardous Event → Consequences → Control Measures → Measure Costs → Risk Reduction → Residual Risk.”

**Figure 2:**
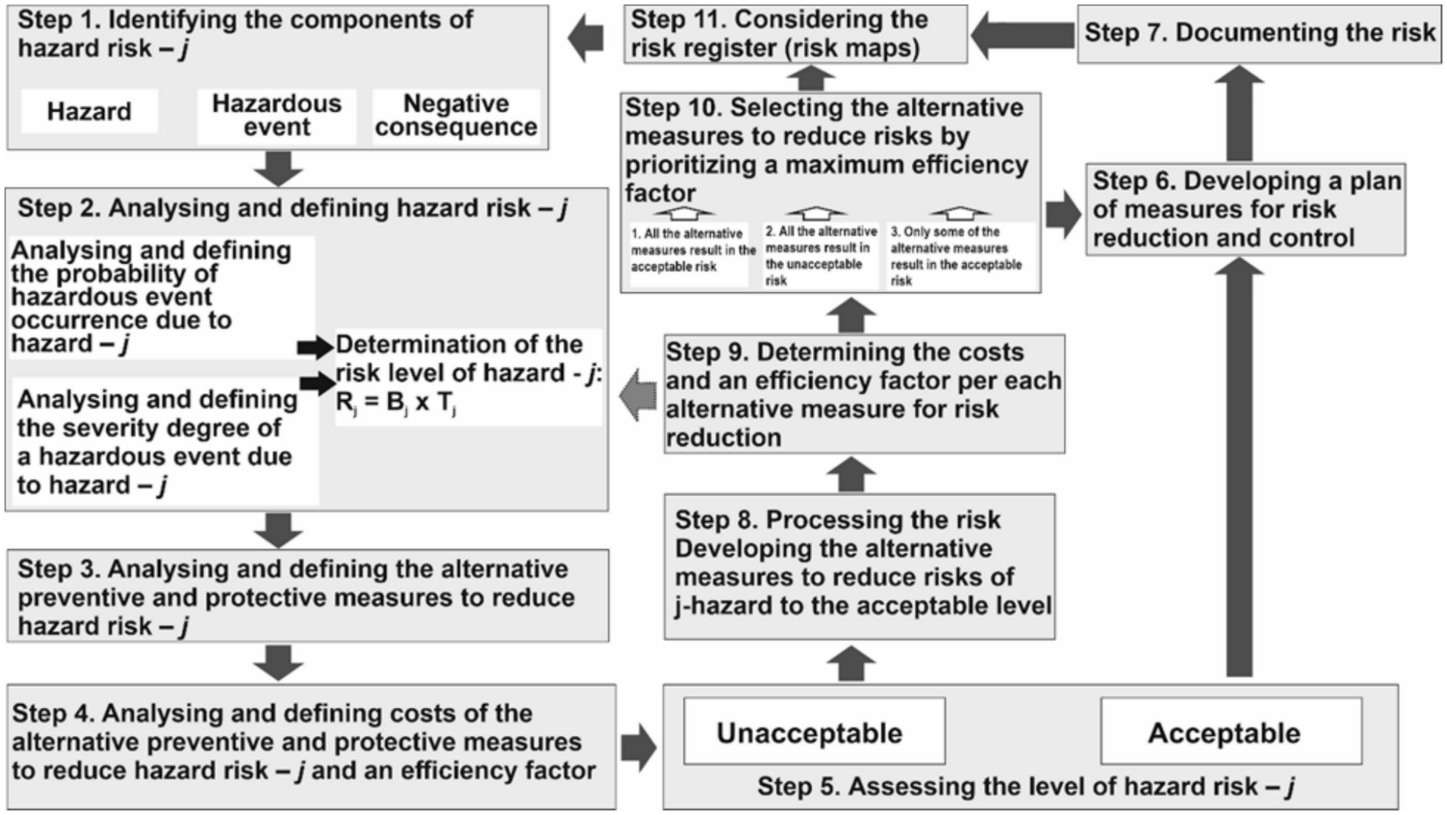
Risk management process for determining risk reduction measures while considering the maximum efficiency ratio in terms of minimizing financial costs.

It is assumed that only those alternatives will be considered technically feasible for implementation under the specific conditions of the enterprise. In other words, measures that are clearly infeasible due to technical constraints should not be included in the list of alternatives for further analysis based on the corresponding criteria (Figure 2).

Step 4 ranks the risk reduction measures based on the efficiency factor calculated using the following formula (Equation 1):


(1) $$K_{\mathrm{ef}}=\frac{\left(R_{\mathrm{ni}}-R_{\mathrm{ki}}\right)}{C_{i}},(\text{points}/\mathrm{UAH})$$

where *R_пi_* is the initial level of hazard risks; *R_ki_* is the residual risk level after applying the *i-*th measure for risk reduction; and *C_i_* is the cost of the *i-*th implementation of the risk reduction measure, UAH.

Step 5 assesses the risk level based on the condition that the acceptable risk was 0–50 points and the unacceptable risk was 51–100 points. A 10 × 10 matrix was proposed for the risk assessment. If there is an acceptable risk level, we develop a plan of measures to reduce the main risks and monitor and control the production process parameters for early detection of inconsistencies in the specified characteristics of equipment, technology, or employee behavior (Step 6). Subsequently, we documented the risks according to the specified form (Table 1) (Step 7).

**Table 1:** Systematization of input information to solve problems of selecting among the alternative measures with minimum costs to reduce risks to an acceptable hazard level.

Parameters		*j*								
Identification	Hazard	*Н_j_*								
	Hazardous event due to hazard	*Н_ej_*								
	Hazardous consequences due to the hazard	*НC_j_*								
Primary analysis and determination of the risk level	Probability of the hazardous event occurrence	*P_j_*								
	Severity degree of the hazardous event consequences	*SD_j_*								
	Risk level due to hazard	*R_j_*								
Primary assessment of risks due to the hazard		+/−								
Measures to reduce risks to an acceptable level	Risk reduction measures	*N*_j1_	*N*_*j*2_	*N*_*j*3_	*N*_*j*4_	*N*_*j*5_	*N*_*j*6_	*N*_*j*7_	…	*N_jm_*
	Costs for preventive and protective measures	*C*_*j*1_	*C*_*j*2_	*C*_*j*3_	*C*_*j*4_	*C*_*j*5_	*C*_*j*6_	*C*_*j*7_	…	*C_jn_*
Secondary analysis and determination of the risk level considering the risk reduction measures	Probability of the hazardous event occurrence	*P*_*kj*1_	*P*_*kj*2_	*P*_*kj*3_	*P*_*kj*4_	*P*_*kj*5_	*P*_*kj*6_	*P*_*kj*7_	…	*P_kjn_*
	Severity degree of the hazardous event consequences	*SD*_*kj*1_	*SD*_*kj*2_	*SD*_*kj*3_	*SD*_*kj*4_	*SD*_*kj*5_	*SD*_*kj*6_	*SD*_*kj*7_	…	*SD_kjn_*
	Residual risk level due to hazard	*R*_*k*1_	*R*_*k*2_	*R*_*k*3_	*R*_*k*4_	*R*_*k*5_	*R*_*k*6_	*R*_*k*7_	…	*R_kn_*
	Risk reduction, ∆*R* = *R_j_* – *R_ki_*	*∆R*_1_	*∆R*_2_	*∆R*_3_	*∆R*_4_	*∆R*_5_	∆*R*_6_	*∆R*_7_	….	*∆R_n_*
Final assessment of the risk considering the risk reduction measures*		+/−	+/−	+/−	+/−	+/−	+/−	+/−	…	+/−
Efficiency of the risk reduction measures		*E*_*j*1_	*E*_*j*2_	*E*_*j*3_	*E*_*j*4_	*E*_*j*5_	*E*_*j*6_	*E*_*j*7_	…	*E_jn_*
Priority of selecting measures to reduce the risk level to an acceptable one		*PS*_*j*1_	*PS*_*j*2_	*PS*_*j*3_	*PS*_*j*4_	*PS*_*j*5_	*PS*_*j*6_	*PS*_*j*7_	…	*PS_jn_*

1. Designation j is the number of current hazards; j = 1, 2, 3,…., k, where k is the number of hazards and m is the number of preventive or protective measures of hazard j; *n* = 1, 2, 3,…. where n denotes the hazard amount for each measure. 2. * +/− corresponds to acceptable/unacceptable risks. 3. While calculating the risk, the following were used: probability from 0 to 1, severity degree of 0–100 points, acceptable risk was 0–50 points, and unacceptable risk was 51–100 points.

In the case of a situation in which the risk level is unacceptable, we proceed immediately to Step 8, where the risk is processed by selecting a certain set of measures to reduce risks, which will allow us to obtain the desired result—an acceptable risk level.

Step 9 estimates the financial cost of their implementation. Next, we set the risk reduction efficiency factor. This helps to prioritize them based on the most effective alternative measure to the least effective alternative measure to reduce risks. If it is impossible to select sufficient risk reduction measures, there is a need to return to Step 2, where the possibility of introducing other risk assessment methods, refining the scales, and testing the hypotheses made at the beginning of the risk management process are considered.

Step 10 determines which risk reduction measures from a set of alternatives are best implemented in the production process to reduce the risk level to an acceptable level. To do this, we solve one (or, if necessary, all) of the three problems of measurement substantiation based on the following conditions.

- Select one measure from the alternatives, each of which reduces the risks to an acceptable level.
- A certain combination of alternative measures allows reducing the risks to an acceptable level.

Select several alternative measures where only some of the protective and preventive measures reduce risks to an acceptable level.

The solution to the proposed problems will make it possible to substantiate risk reduction measures and move on to Step 6 regarding the development of a plan for implementing the relevant measures in the production process.

The last step is creating a register of hazards for further monitoring and revision if necessary.

The proposed risk management process incorporates feedback loops through the possibility of returning from Step 9 (assessment of financial costs and efficiency of the selected measures) to Step 2 (risk analysis and assessment). If, in Step 9, it is determined that the selected preventive measures are either insufficiently effective or too costly to achieve an acceptable risk level, the process returns to the analysis stage to revise the risk assessment methods, consider alternative options, or even reassess the initial assumptions. This iterative cycle ensures adaptability and coordination of the process to achieve the final objective.

The selection of an appropriate alternative for risk control and reduction involves two key criteria applied in Step 10: reducing the risk to an acceptable level (in the given example, to 50 points or lower) and minimizing financial costs.

To achieve this, a clear definition of the system boundaries is required (i.e., the feasibility of applying the alternatives), which encompasses the entire risk management cycle from hazard identification to post-implementation monitoring (Step 11).

The proposed risk management process for selecting alternatives to control risks is based on several key principles: viewing the object as a system of interrelated elements, establishing cause-and-effect relationships, using models to represent system behavior, applying feedback mechanisms for adaptation, and employing clear criteria for making appropriate decisions on risk reduction or control. These principles ensure a logical structure, consistency, and coordination of all steps in the process, ultimately aiming to achieve an optimal (i.e., most cost-effective) reduction of risk to an acceptable level.

To solve the second problem of determining measures to reduce risk from a set of alternatives, it is necessary to generate input data for calculation based on the completion of the eight previous steps.

1. Establish the probability of occurrence of a dangerous event and the degree of consequence severity according to the appropriate scales and risk assessment (steps 1 and 2); to do that, you can use the recommendations given by Luyten et al. (36).
2. Determine the results of the initial assessment of the occupational risk level according to the recommendations of Lutyński and Lutyński (37) (Step 5).
3. Define all measures to reduce risks (Steps 3 and 4), their selection, and risk reduction from the specified hazard (Step 8) to establish financial losses for their implementation (Step 9), in accordance with the recommendations of Fujii et al. (38).

Table 1 systematizes the collected information based on the specified initial data.

According to the risk management process, there is a need to solve the three problems described above. Solving the first one helps find a risk reduction measure among the alternatives to reduce the risk level to an acceptable level. To do so, it is necessary to compare the risk reduction efficiency factors of all the alternative measures and determine their priorities.

## 3 Results and discussion

The level of risk for a certain hazard is 64 points, which is unacceptable. To reduce this, seven measures with corresponding risk reductions and financial costs were established (Table 2).

**Table 2:** Results of selecting a risk reduction measure from a set of alternatives with minimum costs.

Parameters		*j*						
Identification	Hazard	*Н_j_*						
	Hazardous event due to hazard	*НE_j_*						
	Hazardous consequences due to the hazard	*НC_j_*						
Primary analysis and determination of the risk level	Probability of the hazardous event occurrence	0.8						
	Severity degree of the hazardous event consequences	80						
	Risk level due to hazard	64						
Primary assessment of risks due to the hazard		–						
Measures to reduce risks to an acceptable level	Risk reduction measures	*N*_*j*1_	*N*_*j*2_	*N*_*j*3_	*N*_*j*4_	*N*_*j*5_	*N*_*j*6_	*N*_*j*7_
	Costs for preventive and protective measures	1,000	5,000	30,000	5,000	1,000	6,000	900
Secondary analysis and determination of the risk level considering the risk reduction measures	Probability of the hazardous event occurrence	0.7	0.8	0.5	0.4	0.5	0.3	0.2
	Severity degree of the hazardous event consequences	70	60	100	80	30	80	70
	Residual risk level due to hazard	49	48	50	32	15	24	14
	Risk reduction, ∆*R* = *R_j_* – *R_ki_*	15	18	14	32	49	40	50
Efficiency of the risk reduction measures		0.0015	0.0036	0.0005	0.0064	0.049	0.0066	0.0566
Priority of selecting a highly efficient preventive or protective measure to reduce risks to an acceptable level		6	5	7	4	2	3	1

**Figure 3:**
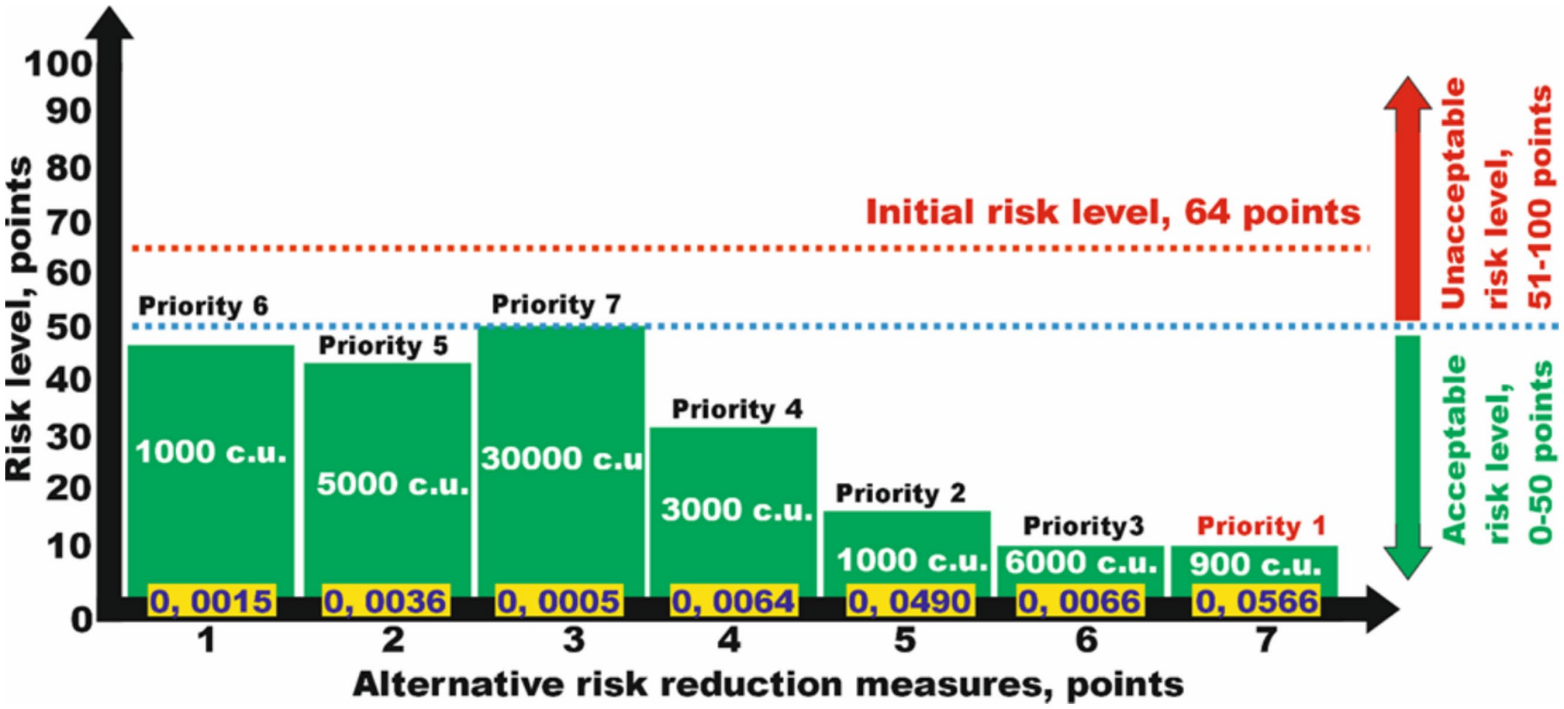
Analysis of the efficiency of risk reduction measures from a set of alternatives to select the one reducing the risks to an acceptable level while considering minimum costs.

It is necessary to define the most expedient option based on the criterion of minimum costs. While analyzing the data in Table 2, we can see that each of the proposed measures can independently reduce the risk to an acceptable level, i.e., reduce the risk level to less than 50 points (Figure 3). When evaluating the priority of measures using the risk reduction efficiency factor, we selected the measure numbered *N*_*j*7_. This reduced the risk to an acceptable level of 14 points with a maximum efficiency factor for risk reduction and a minimum cost of 900 currency units (c.u.).

**Figure 4:**
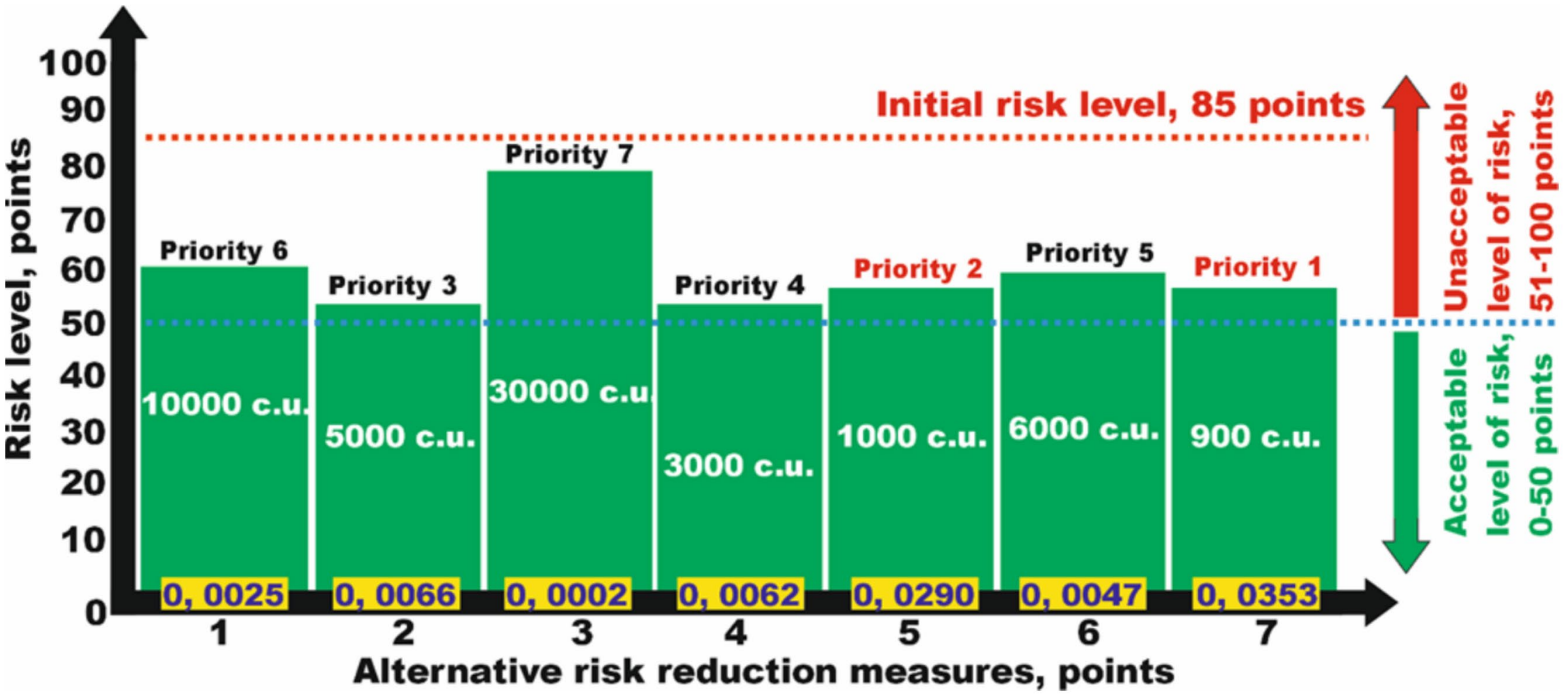
Analysis of the efficiency of risk reduction measures from a set of alternatives to select several ones to reduce risks to an acceptable level while considering minimum financial costs.

The second problem arises when not all alternative measures individually reduce the risk to an acceptable level on their own. In a conditional example, to reduce the risk level from a hazard of 85 points, it was not possible to select one of the seven proposed alternative measures that allowed reducing the risks to an acceptable level (Figure 4).

This makes it necessary to choose several measures to reduce the risk levels. While analyzing the data in Table 3, we select, in accordance with the specified priorities from 1 to 7, several risk reduction measures that together will help obtain the desired result. The specified conditions are satisfied by the measures numbered *N*_*j*5_ and *N*_*j*7_, which together reduce the risk to 28 points (acceptable risk), with a total financial cost of 1.900 c.u. (Table 3), which is significantly lower than that for the implementation of other alternatives.

**Table 3:** Results of the selection of several measures from a set of alternatives, reducing the risks to an acceptable level with minimum costs.

Parameters		*j*						
Identification	Hazard	*Н_j_*						
	Hazardous event due to hazard	*НE_j_*						
	Hazardous consequences due to the hazard	*НC_j_*						
Primary analysis and determination of the risk level	Probability of the hazardous event occurrence	0.9						
	Severity degree of the hazardous event consequences	94						
	Risk level due to hazard	85						
Primary assessment of risks due to the hazard		–						
Measures to reduce risks to an acceptable level	Risk reduction measures	*N*_*j*1_	*N*_*j*2_	*N*_*j*3_	*N*_*j*4_	*N*_*j*5_	*N*_*j*6_	*N*_*j*7_
	Costs for preventive and protective measures	10,000	5,000	30,000	5,000	1,000	6,000	900
Secondary analysis and determination of the risk level considering the risk reduction measures	Probability of the hazardous event occurrence	0.6	0.8	0.8	0.9	0.7	0.6	0.5
	Severity degree of the hazardous event consequences	100	65	100	60	80	95	95
	Residual risk level due to hazard	60	52	80	54	56	57	52.2
	Risk reduction, ∆*R* = *R_j_* – *R_ki_*	25	33	5	31	29	28	31.7
Efficiency of the risk reduction measures		0.0025	0.0066	0.0002	0.0062	0.029	0.0047	0.0353
Priority of selecting a highly efficient preventive or protective measure to reduce risks to an acceptable level		6	3	7	4	2	5	1

The solution to the third problem of selecting measures from the alternative ones is based on their priority, which relies on the risk reduction efficiency factor. For the conditional example, the hazard risk level is 85 points. At the same time, none of the seven proposed measures helped reduce the risk to an acceptable level. In this case, it is proposed to determine the measures from the set of alternatives in the order of priority according to the risk reduction efficiency factor, which will allow reducing the risks to an acceptable level (Table 4).

**Table 4:** Results of selecting the measures from a set of alternatives, provided that only some measures result in the acceptable risk level.

Parameters		*j*						
Identification	Hazard	*Н_j_*						
	Hazardous event due to hazard	*НE_j_*						
	Hazardous consequences due to the hazard	*НC_j_*						
Primary analysis and determination of the risk level	Probability of the hazardous event occurrence	0.9						
	Severity degree of the hazardous event consequences	95						
	Risk level due to hazard	85.5						
Primary assessment of risks due to the hazard		–						
Measures to reduce risks to an acceptable level	Risk reduction measure, risk	*N*_*j*1_	*N*_*j*2_	*N*_*j*3_	*N*_*j*4_	*N*_*j*5_	*N*_*j*6_	*N*_*j*7_
	Preventive and protective measures for risk reduction	10,000	5,000	30,000	5,000	1,000	6,000	900
Secondary analysis and determination of the risk level considering the risk reduction measures	Costs for preventive and protective measures	0.6	0.8	0.8	0.9	0.7	0.6	0.55
	Probability of the hazardous event occurrence	80	65	60	60	80	95	95
	Severity degree of the hazardous event consequences	48	52	48	54	56	57	52.2
	Residual risk level due to hazard	37.5	33.5	37.5	31.5	29.5	28.5	33.2
Efficiency of the risk reduction measures		0.0038	0.0007	0.0013	0.0063	0.0295	0.0048	0.0369
Priority of selecting a highly efficient preventive or protective measure to reduce risks to an acceptable level		5	7	6	3	2	4	1

**Figure 5:**
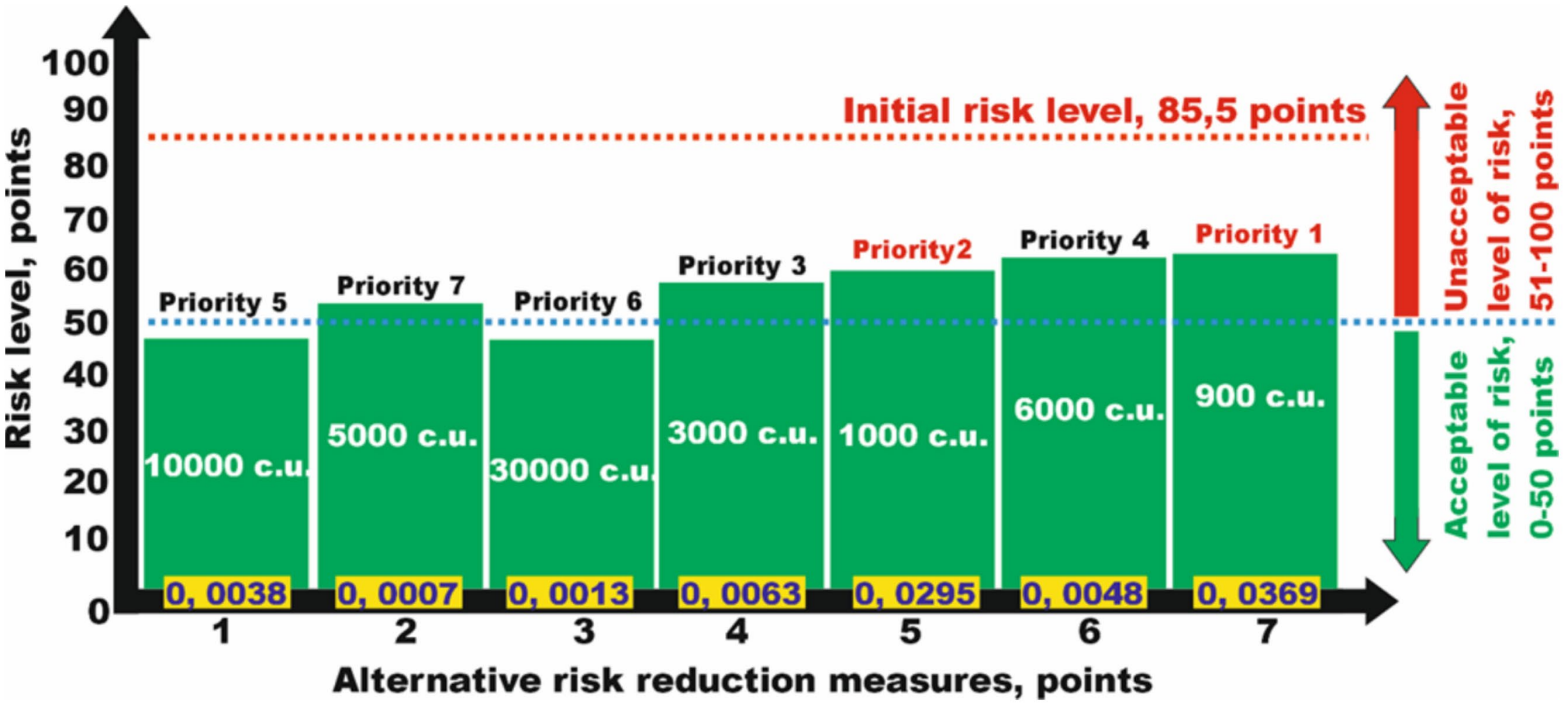
Analysis of the efficiency of risk reduction measures from a set of alternatives for selecting several ones, if only some of them result in an acceptable risk level.

The analysis of determining the measures from a set of alternatives showed that the best option is the selection of measures *N*_*j*5_ and *N*_*j*7_, which allows reducing the risk to 23.25 points (acceptable risk), while the financial costs for their implementation will amount to 6.900 c.u. (Figure 5). Other combinations, such as *N*_*j*1_ and *N*_*j*3_, require significantly more funds at 40.000 c.u. (10.000 and 30.000 c.u., respectively).

To solve the third problem, consider an example of determining measures for risk reduction to an acceptable level from a set of alternatives, provided that their financial costs are minimized in a coal mine. A significant problem in coal mines is the reduction in the number of occupational pneumoconiosis cases among the miners.

To solve this problem, we need to select the effective protective measures for dust reduction in mine workings during rock mass extraction and transportation. Within some mine sites, the coal dust concentration reaches up to 1 g/m^3^ at a permissible dust level, with a silicon oxide content of 2% at a level of 10 mg/m^3^. Usually, to reduce dust in coal mines, the replacement of mining equipment is used (39, 40).

An example is provided to illustrate the determination of preventive or protective measures to reduce the risk of occupational pneumoconiosis in miners to an acceptable level based on a set of alternative dust reduction strategies in coal mines.

Pneumoconiosis among coal miners remains a significant problem that needs to be addressed (41). The most common forms of this disease include silicosis, diffuse fibrosis, emphysema, and chronic bronchitis, all of which may be fatal in severe cases (42). Mining industries in countries such as China (43), India (44, 45), Ukraine (46), and other regions with complex mining and geological conditions are particularly characterized by a high risk of occupational respiratory diseases. In some Chinese coal mines, coal dust concentrations can reach up to 1 g/m^3^, whereas the permissible dust level (RDL) with a crystalline silica content of 5–10% (CFS) is limited to 2.5 mg/m^3^ (47).

The primary causes of pneumoconiosis include the composition of coal dust, its concentration, and the duration of exposure to hazardous zones (48–50). It is difficult to influence the composition of coal dust because it depends on the geological characteristics of the coal seam and properties of the extracted coal (e.g., location, hardness, and degree of coalification). Therefore, the most realistic opportunity to reduce the risk of occupational diseases is to minimize the generation of coal dust through modifications in the mining methods, transportation techniques, and technologies used for coal preparation. Additionally, the time miners spend in hazardous areas can be reduced using modern unmanned mining technologies or remote-control systems (51).

In the proposed example for determining alternative measures to reduce the risk of pneumoconiosis, it was assumed that it is not possible to influence the mining and geological conditions that determine the mineral composition of the coal dust. Therefore, among various engineering options, we focused on methods aimed at reducing the dust concentration. The goal was to demonstrate how one or more alternative preventive measures could be justified to reduce the risk of pneumoconiosis to an acceptable level.

It should be noted that the “acceptable risk level” presented in the example is the conventionally adopted value used here for illustrative purposes. In practice, this threshold is established by each organization individually based on its specific conditions, capabilities, and requirements of national legislation.

The main objective of this example is to determine the feasibility of applying one or more alternative measures to reduce dust concentrations under otherwise equal conditions.

For example, it is proposed to replace the shearer with a plow installation (N1), which allows for a significant reduction in the number of miners required in the stoping area to control the coal mining process. In addition, the plow installation cuts much larger coal pieces than the shearer does, which contributes to significantly less dust formation within the working area. Moreover, there were the following means of dust reduction: intensive face ventilation (*N*_2_), preliminary moistening of the coal seam (*N*_3_), use of Venturi scrubbers on the working body of a shearer/plow (*N*_4_), dust reduction by hydro-irrigation (*N*_5_), and use of personal protective equipment for respiratory organs (*N*_6_). Data on alternative protective/preventive measures, their estimates, and their effectiveness are presented in Table 5.

**Table 5:** Results of selecting the measures from a set of alternatives, provided that only some measures result in an acceptable risk level.

Parameters		1					
Identification	Hazard	Coal dust					
	Hazardous event due to hazard	Pneumoconiosis					
	Hazardous consequences due to the hazard	Disability, death due to lung fibrosis					
Primary analysis and determination of the risk level	Probability of the hazardous event occurrence	0.9					
	Severity degree of the hazardous event consequences	95					
	Risk level due to hazard	85					
Primary assessment of risks due to the hazard		Unacceptable					
Measures to reduce risks to an acceptable level	Reduction measure, risk	*N*_1_	*N*_2_	*N*_3_	*N*_4_	*N*_5_	*N*_6_
	Risk reduction measure, risk	20 million	504 thousand	352 thousand	220 thousand	150 thousand	750 thousand
Secondary analysis and determination of the risk level considering the risk reduction measures	Preventive and protective measures for risk reduction	0.6	0.8	0.8	0.9	0.7	0.6
	Costs for preventive and protective measures	80	65	60	60	80	95
	Probability of the hazardous event occurrence	48	52	48	54	56	57
	Severity degree of the hazardous event consequences	37.5	33.5	37.5	31.5	29.5	28.5
Efficiency of the risk reduction measures		2.12	6.61	2.73	1.42	1.13	1.82
Priority of selecting a highly efficient preventive or protective measure to reduce risks to an acceptable level		5	7	6	3	2	4

The 85-point assessment of pneumoconiosis risk in miners, which is unacceptable at the permissible limit of 50 points, demonstrates the need to identify the best alternative. While analyzing the data in Table 5, we can see that only two protective measures from the proposed alternatives can reduce the risks independently to an acceptable level (less than 50 points). When evaluating their priority according to the efficiency factor, we select alternative number *N*_3_—preliminary coal seam moistening, which will reduce the risk to 47 points, with the maximum efficiency factor of 1.13 points and minimum financial costs of 352,000 c.u.

In this study, the main focus was on developing a methodological approach for selecting alternative measures to reduce risks to an acceptable level. To simplify and better illustrate the decision-making process, the following assumptions were made: Specifically, the “implementation cost of a measure” includes only the direct expenses related to its realization (e.g., the cost of equipment procurement, installation, initial training, etc.).

Moreover, the time required to implement preventive measures is not considered in this analysis. It is assumed that time can be incorporated as an additional decision-making criterion in future applications. For example, when measures have similar cost indicators, preference may be given to those with a shorter implementation time. Alternatively, time can be integrated into a modified version of the efficiency calculation formula in subsequent studies.

The introduction of new approaches to the control of occupational safety and health of workers based on risk-oriented thinking requires determination of their efficiency. At the same time, the available approaches based on the accumulation of statistical data on violations of safety instructions, the number of accidents, days of working incapacity, and the severity of injuries often fail to provide comprehensive feedback on the implementation results of new labor safety measures. It is since these indicators focus on the events that have already happened (52, 53).

After identifying relevant risks in the organization, authorities should plan actions to eliminate them with the help of measures, including, among other things, the substantiation of costs. There is a need to choose an option that will allow the best response to the manifestation of risks to reduce negative consequences, with minimum costs for preventive/protective measures (54, 55). In any organization, its top management must make decisions on risk analysis and must be constantly attentive to changes in the external context, being able to manage risks by keeping them at an acceptable level with minimum costs for preventive/protective measures (32, 56).

Economic evaluation can be a valuable tool for occupational safety and health decision-makers dealing with resource allocation tasks, as it provides detailed information on the costs and outcomes of an intervention. When done carefully, it provides useful estimates of the investment profitability of competing programs. However, several studies have indicated that published studies are often insufficiently robust, and better economic evaluation studies are required (57).

The problems of economic assessment of implemented solutions in the field of labor safety are practically limited to the three tasks discussed above. Their implementation is a complex process that often requires creative methodological approaches. An expert opinion on the efficiency of certain preventive/protective measures is also important. This allows you to obtain different points of view, helping avoid different cognitive biases regarding the selection of the best option (49, 58). Therefore, it is necessary to develop appropriate models that will allow consideration of the timeframe for the introduction of preventive/protective measures, as well as the influence of biases on managerial decision-making.

## 4 Conclusion

A risk management process was developed with the selection of measures from a set of alternatives, taking into account the risk reduction efficiency of the measure in terms of minimizing financial costs.

The selection of measures from a set of alternatives based on the eleven-step process of risk management has been substantiated. Priority was considered according to the risk reduction efficiency factor. Factor specificity is the consideration of three problems (typical cases): problem 1 involves selecting a measure from a set of alternatives, where each alternative measure reduces the risks to an acceptable level; problem 2 involves selecting several measures from a set of alternatives that do not reduce the risks independently to an acceptable level; and problem 3 involves selecting a combination of measures from a set of alternatives, where only some measures reduce the risks to an acceptable level.

It has been determined that preliminary moistening of coal seams is rather an effective measure among the proposed protective measures to reduce pneumoconiosis risks in miners.

## Data Availability

The original contributions presented in the study are included in the article/supplementary material, further inquiries can be directed to the corresponding author.
